# High-Frequency Dynamics and Electrical Signatures of a 3D Bloch Point

**DOI:** 10.3390/nano16161005

**Published:** 2026-08-15

**Authors:** Zukhra Gareeva, Shamil Gareev, Viktoria Filippova, Ildus Sharafullin

**Affiliations:** 1 Institute of Molecule and Crystal Physics, Subdivision of the Ufa Federal Research Centre of the Russian Academy of Sciences, 450075 Ufa, Russia; 2 Institute of Physics and Technology, Ufa University of Science and Technology, 450000 Ufa, Russia

**Keywords:** magnetic nanostructures, Bloch point, high-frequency spintronics

## Abstract

Three-dimensional topological magnetic defects, such as Bloch points, are of significant interest for high-frequency spintronics due to their unique particle-like properties and effective inertial mass. We investigate the nucleation, stabilization, and driven dynamics of an isolated Bloch point in a ferromagnetic multilayer with alternating in-plane and perpendicular magnetic anisotropy. Using micromagnetic simulations, we show that a perpendicular magnetic field stabilizes a head-to-head Bloch point state, while a transient in-plane field pulse drives the defect into gyrotropic and nutation motion. To model the dynamics, we develop a collective-coordinate Lagrangian description of the Bloch point core. We further demonstrate that the time-dependent core displacement generates a transverse charge current via spin pumping and inherent spin-to-charge conversion within the multilayer system. The resulting current spectrum contains low-frequency and high-frequency components, including an intrinsic nutation mode in the gigahertz range. Our findings expand the capabilities for electrical control and identification of complex spin configurations, contributing to the development of active three-dimensional spintronic devices.

## 1. Introduction

The rapid progress in information technology demands novel functional materials and device architectures capable of operating at sub-100 nm scales. While conventional electronics encounter fundamental limits, spintronics offers alternative pathways by exploiting the electron’s spin degree of freedom [1,2,3,4]. Reflecting this potential, the global spintronics market is projected to expand significantly, reaching over USD 3.5 billion within the next decade, driven by demand for energy-efficient MRAM architectures, ultra-sensitive field sensors, and high-frequency spintronic components. Beyond conventional memory applications, dynamic spin textures and their high-frequency transport signatures offer promising pathways for quantum-assisted technologies and bio-spintronics, may find applications in hybrid spintronic sensing platforms employing molecular spin systems.

The continued demand for faster, energy-efficient, and highly scalable information-processing technologies therefore motivates the search for new three-dimensional magnetic states with enhanced functionality and reliable electrical readout mechanisms. In this context, three-dimensional (3D) topological magnetic defects hosted within localized nanostructures have recently attracted significant attention due to their unique particle-like properties, enhanced stability, and potential for vertical information transport [5,6,7,8,9].

A Bloch point (BP) is a zero-dimensional singularity in the magnetization field, where the local magnetic moment is undefined and the surrounding magnetization assumes all possible spatial orientations within a finite volume [10,11,12,13,14]. First predicted by Feldtkeller and Doering [10,15] and later inferred from domain wall dynamics in garnet films [16,17,18], BPs have become the subject of intensive research owing to advances in high-resolution imaging techniques, such as Lorentz transmission electron microscopy and X-ray holography, which have enabled their direct visualization [7,9,19,20]. Beyond serving as core nucleation and annihilation centers in skyrmionics, BPs offer viable pathways for ultrafast magnetization reversal at the nanoscale [21,22,23].

While the static properties of BPs have been extensively investigated in bulk chiral magnets, nanopillars, and nanowires [7,12,14,24,25,26], multilayer films provide a highly controlled platform for their stabilization. By stacking layers with different Dzyaloshinskii–Moriya interactions (DMI) and competing magnetic anisotropies, the BP core radius, nucleation field, and stability range can be finely tuned via intrinsic material parameters and interlayer exchange coupling [27,28].

However, the practical utilization of BPs in devices is currently hindered by a critical bottleneck: the lack of an efficient electrical readout mechanism. Standard methods like anisotropic magnetoresistance and the topological Hall effect yield weak electrical signals, particularly for 3D objects where the interaction volume is extremely small [29,30]. To overcome this limitation, alternative mechanisms exploiting magnetization dynamics have been proposed. Specifically, the inverse spin Hall effect and spin-to-charge conversion can efficiently convert time-varying magnetization into charge currents via spin–orbit coupling [29,30,31,32,33]. While this approach has been successfully employed to detect domain wall motion and skyrmion dynamics, or to generate magnetogalvanic currents in conducting antiferromagnets (e.g., CuMnAs and Mn_2_Au) [30,34,35,36], an analogous mechanism for ferromagnetic systems hosting 3D topological defects has not yet been established. Consequently, a key question is whether the dynamic displacement of a BP core can be efficiently converted into a measurable electrical signal.

In this paper, we address this question by establishing a link between the static BP configuration and its dynamical magnetotransport response. We first present micromagnetic simulations of BP nucleation and stabilization in the layered ferromagnetic structure, quantifying the core parameters. We then develop an analytical Lagrangian model describing the BP core dynamics driven by a time-dependent magnetic field pulse. Within this framework, we demonstrate that the moving BP core acts as a source of coherent spin precession, generating a measurable current via dynamic spin-to-charge conversion. The resulting electrical signal exhibits a characteristic multi-frequency spectrum, including a low-frequency gyrotropic mode, a forced oscillation at the driving frequency, and a high-frequency nutation peak associated with the BP inertial mass.

The paper is organized as follows. Section 2 presents the micromagnetic model and simulation parameters, Section 3 describes the magnetization reversal processes that lead to the stabilization of the Bloch point and Section 4 introduces the Lagrangian model of the Bloch point core dynamics. Section 5 analyzes the high-frequency electrical response. Finally, concluding remarks are summarized in the Conclusion section.

## 2. Methods and Materials

To investigate the localized dynamic excitations of a three-dimensional topological defect, it is first necessary to establish a stable, isolated Bloch point (BP) configuration as a baseline state. While BPs in bulk crystals often require specific constraints or strong continuous external biases, multi-layered ferromagnetic films offer a highly controllable alternative for stabilizing such defects.

In this work, we consider a model multilayer system composed of alternating layers with in-plane magnetic anisotropy (IMA) and perpendicular magnetic anisotropy (PMA), structured as an IMA/PMA/PMA/IMA stack. As a material platform for hosting and stabilizing such topological magnetic states, we employ rare-earth iron garnet films (M_3_Fe_5_O_12_, where M = Y or rare-earth ions). The garnet crystal structure offers exceptional chemical flexibility; rare-earth substitutions at the M-site enable broad and precise engineering of both the magnetic anisotropy and the orthogonal IMA/PMA transitions [11,37]. Furthermore, recent observations confirm the presence of interfacial Dzyaloshinskii–Moriya interaction (DMI) in RIG heterostructures [38,39], making them an ideal playground for complex 3D spin textures. The choice of this specific multilayer configuration is motivated by two key factors: (i) the competition between the orthogonal anisotropies, interlayer exchange coupling, and demagnetizing fields naturally forces the magnetization to tilt, forming a smooth 3D spin twisting that accommodates a singular BP core, (ii) this configuration ensures that after the external field is removed, the stabilized BP remains strictly isolated and decoupled from other topological defects (such as skyrmion tubes or chiral bobbers), providing a clean environment to quantify the intrinsic inertial and dynamic properties of the 3D core. Note that similar behavior can also be expected in other multilayer architectures with comparable magnetic parameters.

To determine the exact static parameters of the configuration, micromagnetic simulations were performed using the Object Oriented Micromagnetic Framework (OOMMF) software [40], solving the Landau–Lifshitz–Gilbert (LLG) equation

(1) $$\frac{dM}{dt}=-\left|\gamma \right|\left[M\times H_{eff}\right]+\frac{\alpha }{M_{s}}\left[M\times \frac{dM}{dt}\right]$$

where *γ* is the gyromagnetic ratio, *α* is the Gilbert damping constant, *M*_s_ is the saturation magnetization, and $H_{eff}=-\frac{\delta F}{\delta M}$ is the effective field derived from the total free energy functional.

(2) $$F=\sum_{i=1}^{4}\iint dV(A_{i}{\left(\partial_{\mu }m_{i\alpha }\right)}^{2}-K_{i}m_{iz}^{2}-\frac{1}{2}M_{s}m_{i}\cdot H_{m}-M_{s}m_{i}\cdot H-J_{k}m_{k}m_{k+1}+F_{me}+F_{el})$$

where *A_i_* is the exchange stiffness, *α*, *μ* = *x*, *y*, *z*; *K_i_* is the constant of magnetic anisotropy in the *i*-th layer (*K*_1,4_ < 0, *K*_2,3_ > 0); *J_k_* is the interlayer exchange coupling; **H**_m_ is the magnetostatic field; and $m_{i}=M_{i}/M_{s}$ is the unit magnetization vector in the *i*-th layer.

The simulation baseline parameters were chosen as follows: saturation magnetization *M_i_* = 50 kA/m, *A*_1,4_ = 2.9·10^−12^ J/m, *A*_2,3_ = 4·10^−12^ J/m, *K*_1,4_ = −7·10^5^ J/m^3^, *K*_2_ = 2·10^5^ J/m^3^, *K*_3_ = 2·10^3^ J/m^3^, *J*_12,34_ = 3.5·10^−12^ J/m, and *J*_23_ = 0.2·10^−12^ J/m. The nanoelement dimensions are $L_{x}\times L_{y}\times L_{z}=200\times 200\times 96{\;\mathrm{nm}}^{3}$, discretized using a finite-difference grid of $5\times 5\times 3{\;\mathrm{nm}}^{3}$, which is strictly below the material’s exchange length $l_{ex}=\sqrt{\frac{2A}{\mu_{0}M_{s}^{2}}}$. The influence of interfacial rugosity on the BP stability can be estimated by comparing the characteristic roughness amplitude to the exchange length (8.4 nm) and the layer thickness (24 nm per layer). For the garnet system considered here, typical root-mean-square roughness values are below 0.5 nm for high-quality epitaxial films [37], which is an order of magnitude smaller than both the exchange length and the layer thickness. Such roughness is not expected to significantly affect the BP core radius or the dynamic response, although the study of interface disorder remains an interesting avenue for future work. The system is initialized in a uniformly magnetized state with all layers saturated along −*z* and then swept quasi-statically to positive saturation with a convergence criterion ∣*dm*/*dt*∣ < 0.01 deg/ns. We note that the micromagnetic continuum LLG formalism assumes smooth spatial variations of magnetization. Since the effective exchange length and Bloch point core radius Δ=10 nm span several crystal unit cells (garnet lattice parameter *a*_0_ ~ 1.2 nm), atomistic discretization errors remain negligible, validating the continuum LLG framework at these nanometer scales.

## 3. Magnetization Reversal and Nucleation of the Bloch Point

**Figure 1:**
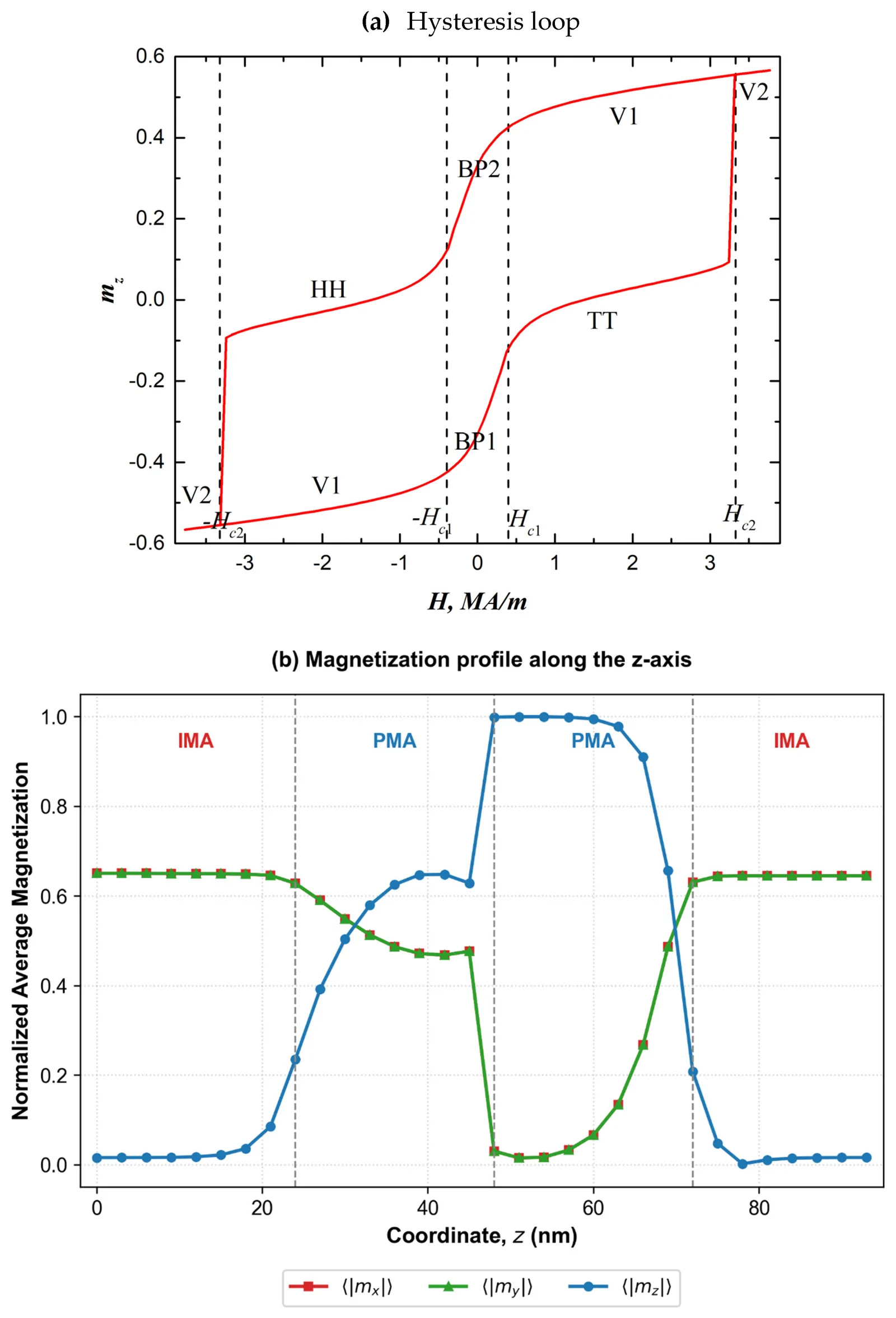

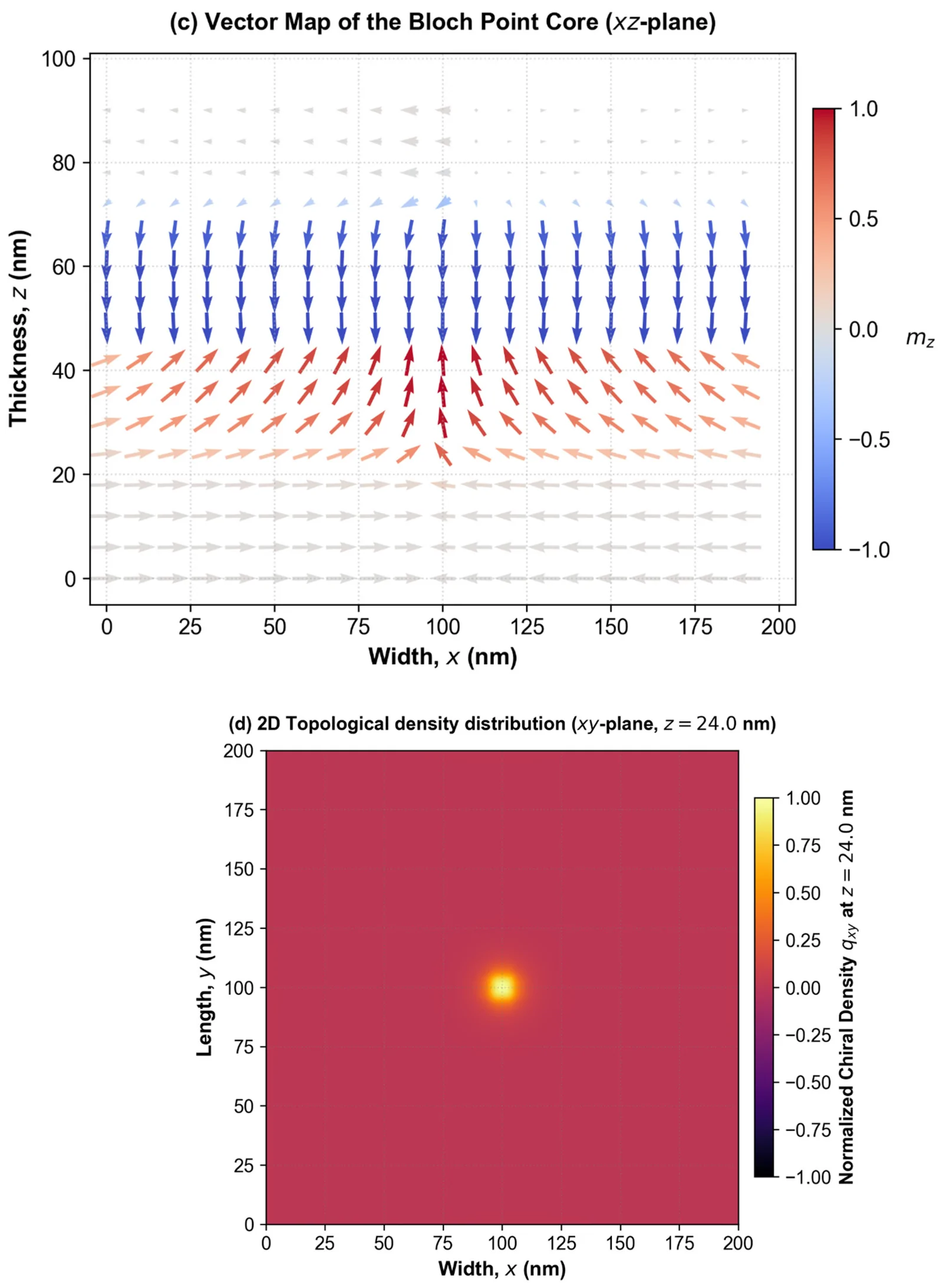

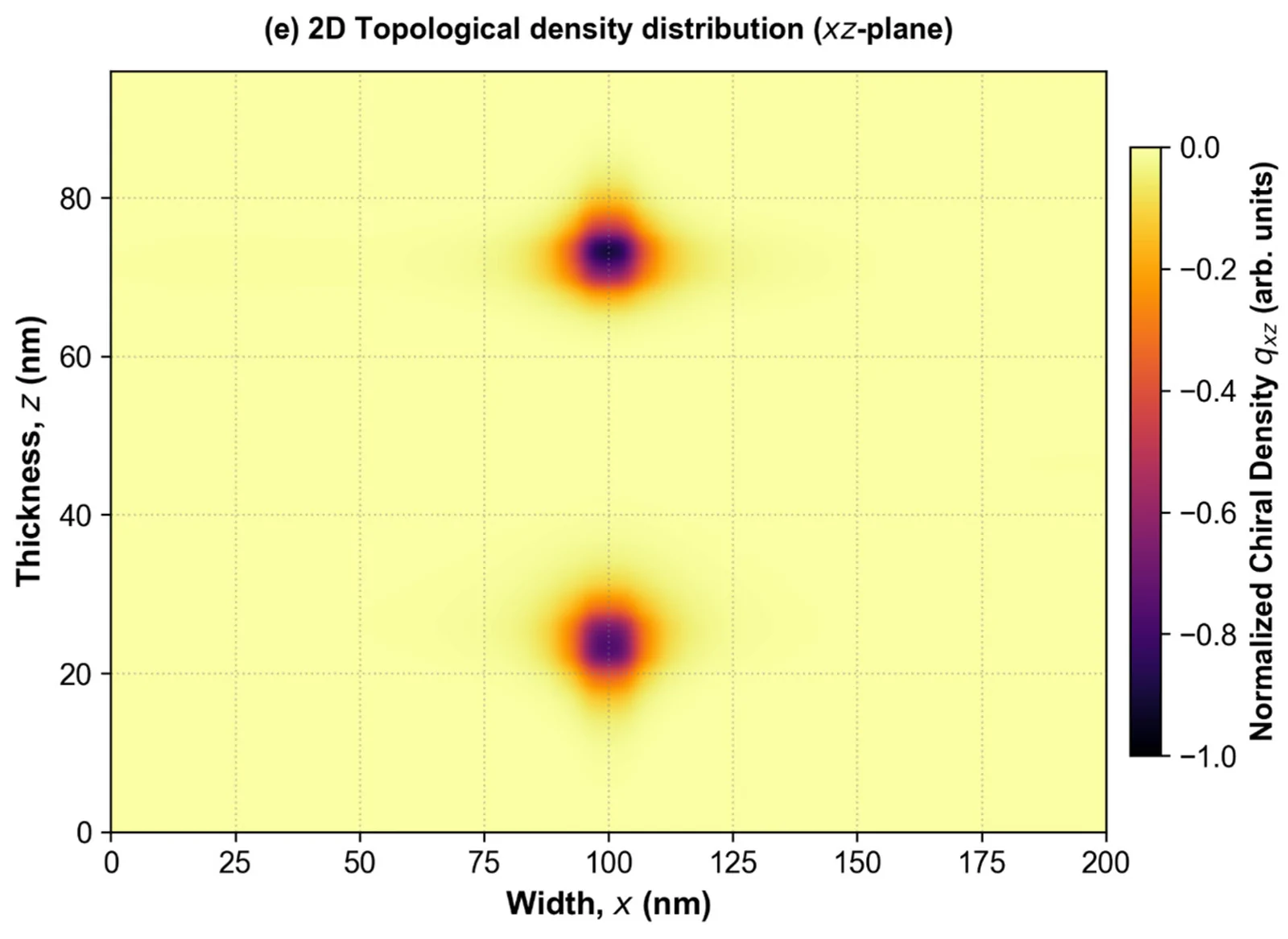
(**a**) Magnetization hysteresis loop m_z_(H) displaying the emerging magnetic phases. (**b**) Spatial depth profile of the normalized average magnetization components across the layer thickness (*z*-axis), (**c**) Cross-sectional vector map of the magnetization in the xz-plane, (**d**) Simulated 2D distribution of the normalized topological density q_xy_ in the vertical central slice (y = 100 nm), showing two symmetric localization zones at the multilayer interfaces. (**e**) Horizontal cross-section of the normalized planar topological density q_xz_ evaluated at the lower PMA interface (z = 24 nm), demonstrating a sharply localized, symmetric vortex core distribution. All color scales in (**c**–**e**) are normalized to the range [−1, 1].

The magnetization reversal process proceeds through a sequence of distinct topological phases. Figure 1a presents the simulated hysteresis loop (the average out-of-plane magnetization component *m_z_* as a function of the applied field *H*) for *K*_2_ = 2 × 10^5^ J/m^3^ and *K*_3_ = 2 × 10^3^ J/m^3^. At large negative fields, the system is in a uniformly magnetized state. As the field approaches zero, the outer IMA layers develop a vortex-like configuration, while the inner PMA layers form a “head-to-head” spin arrangement at their interface. This sharp magnetization gradient is compressed with increasing field until it collapses into a stable BP state at a critical nucleation field *H_c_* ≈ 300 kA/m. The BP remains stable over a finite field interval around *H_z_* = 0, making it suitable for further dynamic investigations.

The spatial structure of the stabilized BP is shown in Figure 1b,c. Near the singularity core, the magnetization can be described by the spherically symmetric ansatz

(3) m(r)=(sinψ(r)cosΦ(r),sinψ(r)sinΦ(r),cosψ(r))

with the radial profile function satisfying the boundary conditions ψ(0)=π, ψ(∞)=0

(4) $$\psi (r)=\pi \left(1-\frac{r}{\sqrt{r^{2}+\Delta^{2}}}\right)$$

where r the distance from the core center measured in the film plane, Δ is the effective core radius, and Φ is the azimuthal angle. From the micromagnetic simulations, we extract Δ ~ 10 nm, consistent with the exchange length.

To further confirm the topological nature of the observed state, we compute the two-dimensional topological charge density distributions *q_xy_* (*x*,*y*), *q_xz_* (*x*,*z*), shown in Figure 1d,e. The localized peak at the core center confirms the presence of a point-like singularity with a well-defined topological signature, consistent with the BP ansatz. The extracted static parameters (Δ ~ 10 nm, *H_c_* ~ 300 kA/m) will be used for the dynamic transport model analyzed in the next section.

To quantify the topological properties of the stabilized magnetic configuration, we evaluate both the local chiral density distributions and the total topological invariants. In any horizontal xy-plane, the local distribution of the skyrmion-like topological charge density is defined as $q_{xy}=\frac{1}{4\pi }m\cdot \left[\frac{\partial m}{\partial x}\times \frac{\partial m}{\partial y}\right]$. Integrating this density over the nanoelement cross-section yields the planar topological charge (vortex core polarity/skyrmion number) $Q_{xy}=\iint q_{xy}dxdy$. Due to the layered IMA/PMA/PMA/IMA architecture, the spatial twisting is non-uniform along the thickness (*z*-axis). On the internal interfaces (*z* = 24 nm and *z* = 72 nm), where the planar vortex configuration matches the perpendicular domain structures, the out-of-plane spin twisting is maximal, leading to pronounced localized peaks in the cross-sectional chiral density (Equation (3)), as shown in Figure 1c. Conversely, in the central symmetry plane (z = 48 nm), the localized distribution of *q_xy_* vanishes due to the purely radial head-to-head configuration of the singular core. The complete three-dimensional topological charge of the system, which confirms the existence of an isolated Bloch point singularity, is invariant and satisfies the mapping condition

$$Q=\frac{1}{4\pi }\iint_{S}q_{ij}dS_{k}=\pm 1$$

where *S* is a closed surface enclosing the core. This non-zero topological invariant generates a powerful effective gyroscopic field that governs the subsequent dynamic excitations of the core under high-frequency driving.

## 4. Dynamic Properties and Current Response

In this section, we bridge the static phase characterization with the dynamic transport properties of the system. In the static case, the out-of-plane magnetic field *H_z_* acts as a control parameter to nucleate and energetically stabilize the Bloch point (BP) configuration. Once the static BP state is firmly established within the robust field interval around zero (*H* ~ 0), this perpendicular configuration forms a parabolic pinning potential well for the singularity core. To investigate the dynamic transport response, we introduce a transient in-plane magnetic field pulse *H_x_*(*t*) as a driving perturbation. This in-plane pulse breaks the axial symmetry, driving the collective coordinates of the BP center ***R*** = (*X*,*Y*) out of its equilibrium position within the film plane. Consequently, while the perpendicular field maintains the topological integrity of the 3D spin defect, the in-plane excitation triggers the complex precessional and nutational trajectories.

### 4.1. Lagrangian Formalism and Thiele Equations of Motion

To describe the motion of a three-dimensional topological defect such as a Bloch point (BP), we employ the collective coordinate approach within the framework of the generalized Thiele-like equations [16,41,42]. This approach allows us to project the continuous field dynamics onto the motion of the singularity center, where the collective dynamic response of the core is governed by the volume-integrated topological invariants.

The dynamics of the BP core are derived from the kinetic Berry phase Lagrangian combined with the Rayleigh dissipation function, accounting for both the intrinsic gyrotropic behavior and the inertial properties of the spin texture. The effective Lagrangian of the system is written as

(5) $$L=G\cdot \left(R\times \dot{R}\right)+\frac{1}{2}m^{*}{\dot{R}}^{2}-U\left(R\right),$$

where $R=\left(X,Y\right)$ defines the collective coordinates of the BP center in the film plane. The first term is the gyrotropic term, which arises from the Berry phase of the magnetization field integrated over the core volume

(6) $$G=QG_{0}z,$$

is the gyrovector, where Q=±1 represents the effective net topological charge acting on the collective coordinates of the center, and ***z*** is the unit normal to the film plane. The second term in Equation (5) represents the effective inertial mass $m^{*}$, which originates from the internal dynamics of the non-collinear magnetization texture accompanying the translational motion of the topological singularity. When the Bloch point moves with an instantaneous translation velocity $\dot{R}$ and acceleration $\ddot{R}$, the non-collinear spin texture undergoes a dynamic distortion relative to its static equilibrium profile. Considering a small displacement of the equilibrium magnetization distribution, the dynamic contribution associated with the deformation of the magnetic texture is determined by the spatial variation of the equilibrium profile. The corresponding effective mass can be written in the general form

(7) $$m_{ij}^{*}=\frac{M_{s}^{2}}{\gamma^{2}}\int_{V}\sum_{k,l}\chi_{kl}\left(\frac{\partial m_{0}^{k}}{\partial X_{i}}\right)\left(\frac{\partial m_{0}^{l}}{\partial X_{j}}\right)dV$$

where *γ* is the gyromagnetic ratio, *∂m*_0_/∂*X_i_* describes the deformation of the equilibrium Bloch-point texture under translation along the *i*-th coordinate, and *χ_kl_* describes the linear response of the transverse spin excitations surrounding the core. For a spherically symmetric 3D Bloch point in an isotropic or weakly anisotropic environment, the off-diagonal mass components vanish ($m_{xy}^{*}=m_{yx}^{*}=0$), rendering the scalar effective mass $m^{*}=m_{xx}^{*}=m_{yy}^{*}$. The potential energy of the core includes anisotropy, magnetostatic energies and the energy of the Zeeman interaction.

(8) $$U\left(R\right)=U_{0}+\frac{1}{2}\kappa \left(X^{2}+Y^{2}\right)-\mu_{eff}H_{x}(t)Y$$

$$\kappa =\kappa_{0}-M_{s}H_{z}\int \frac{\partial^{2}m_{0z}}{\partial y^{2}}dV,\;\mu_{eff}=-M_{s}\int \frac{\partial m_{0x}}{\partial y}dV$$

Here, we consider a transient in-plane magnetic field pulse

(9) $$H_{x}(t)=H_{0}\sin(\omega_{ext}t)\exp(-\beta t).$$

To derive the driving force exerted by this pulse in Equation (9), we employed the rigid-shift approximation (see Appendix A), in which the dynamic magnetization profile is assumed to preserve its shape while translating with the core collective coordinates

(10) $$m\left(r-R\right)=m_{0}\left(r-R\right),$$

where $R\left(t\right)=\left(X\left(t\right),Y\left(t\right),0\right)$ defines the instantaneous position of the Bloch point center.

For small displacements, the Taylor expansion of the magnetization profile relative to the equilibrium state yields $m\left(r-R\right)\approx m_{0}\left(r\right)-X\frac{\partial m_{0}\left(r\right)}{\partial x}-Y\frac{\partial m_{0}\left(r\right)}{\partial y}+\ldots$. Substituting the *x*-component of this expansion into the functional, $U_{H_{x}}=-\mu_{0}M_{0}\int dV(m_{x}\cdot H_{x}(t))$, and exploiting the spatial symmetry of the head-to-head Bloch point core, the coordinate-dependent portion of the potential simplifies to

$$U_{H_{x}}=-\mu_{eff}H_{x}(t)Y,\;\mu_{eff}=\mu_{0}M_{0}\int dV\frac{\partial m_{0x}}{\partial y}.$$

The Euler–Lagrange equations read

$$\frac{d}{dt}\left(\frac{\partial L}{\partial {\dot{X}}_{i}}\right)-\frac{\partial L}{\partial {\dot{X}}_{i}}+\frac{\partial R}{\partial {\dot{X}}_{i}}=0$$

where $R=\frac{1}{2}\hat{D}{\dot{R}}^{2}$ is the Rayleigh function that accounts for the dissipative processes. The effective damping coefficient *D* scales directly with the isotropic components of the Rayleigh dissipation tensor $D_{xx}=D_{yy}=D$, which is calculated via the spatial integration of the magnetization gradients weighted by the Gilbert damping parameter *α*

$$D=\alpha \frac{M_{0}}{\gamma }∭dV\left(\frac{\partial m}{\partial x}\cdot \frac{\partial m}{\partial x}\right).$$

Applying the Euler–Lagrange equations yields the coupled system of second-order differential equations of motion

(11) $$\begin{array}{l} m^{*}\ddot{X}-2QG_{0}\dot{Y}+D\dot{X}+\kappa X=0\\ m^{*}\ddot{Y}+2QG_{0}\dot{X}+D\dot{Y}+\kappa Y=\mu_{eff}H_{x}(t) \end{array}$$

To estimate the effective inertial mass for the localized Bloch-point texture, we reduce Equation (7) to a scaling relation

(12) $$m^{*}\;\sim \;C\frac{M_{s}^{2}R_{BP}}{\gamma^{2}A}$$

by parameterizing the spatial spin profile in spherical coordinates near the core (${\left(\nabla m_{0}\right)}^{2}\;\sim \;1/r^{2}$), and noting that the χ scales inversely with the local exchange energy density. Here *R_B_*_P_ is the characteristic radius of the Bloch-point core, *A* is the exchange stiffness, and $C=\int_{V}\sum_{k,l}\chi_{kl}\left(\frac{\partial m_{0}^{k}}{\partial x/R_{BP}}\right)d^{3}\left(\frac{r}{\partial R_{BP}}\right)$ is a dimensionless coefficient determined by the spatial magnetization profile. For an unperturbed spherically symmetric Bloch-point texture, analytical integration yields $C=\frac{8\pi }{3}$. In constrained multilayer geometries or in the presence of perpendicular magnetic anisotropy, *C* varies within the range of 1–10 depending on film thickness and boundary conditions. This relation demonstrates that the effective inertial mass is a collective property of the entire magnetic texture rather than an intrinsic property of the Bloch-point singularity itself. In particular, it depends on the Bloch-point size, exchange stiffness, multilayer confinement geometry, anisotropy, and interlayer exchange coupling, which define the spatial structure of the non-collinear magnetization surrounding the singularity. For the present multilayer system, the equilibrium Bloch-point configuration obtained from micromagnetic simulations provides the parameters required for estimating the inertial response. Using the calculated core radius *R_BP_* = 10 nm, the exchange stiffness is *A* = 4 × 10^−12^ J/m and the saturation magnetization is *M_s_* = 50 kA/m, we estimate an effective inertial mass $m^{*}\approx 1.08\times 10^{-24}\;kg$, which is subsequently used as the inertial parameter in Equation (11). Variations of the multilayer geometry, magnetic anisotropy profile, interlayer coupling strength, or DMI strength modify the Bloch-point structure and consequently change the value of $m^{*}$. In contrast, within the linear-response regime considered here, the excitation amplitude produces only small displacements of the core and weak distortions of the magnetization profile. Therefore, the effective mass can be considered approximately constant. At larger excitation amplitudes, coupling between translational motion and internal deformation modes may result in an amplitude-dependent effective mass, which requires a full micromagnetic eigenmode analysis beyond the scope of the present work.

Using the obtained value of $m^{*}$, Equation (11) is solved numerically. The finite value of the inertial mass introduces an additional high-frequency eigenmode into the BP dynamics. In the limit of vanishing inertia ($m^{*}$ → 0), Equation (11) reduces to the conventional first-order Thiele equation and the nutation mode disappears. Therefore, the observation of the high-frequency resonance in the electrical spectrum provides direct evidence of the inertial contribution associated with the Bloch-point dynamics. We introduce the dimensionless time $\tau =\omega_{0}t$ scaled by the reference frequency, $\omega_{0}=\kappa /G_{0}$, which maps the gyroconstant to unity (*G* = 1). Under this transformation, the dimensionless parameters scale as $\tilde{m}=\frac{m^{*}\omega_{0}}{G_{0}};\;\tilde{D}=\frac{D}{G_{0}},\;\tilde{\kappa }=\frac{\kappa }{G_{0}\omega_{0}}$. Using the micromagnetic baseline parameters (*M_s_* = 50 kA/m, $L_{z}=96\;nm$, R = 100 nm), we evaluate the physical coefficients $G_{0}\approx 2.73\times 10^{-14}\;J\cdot s/m^{2},$ $D\approx 7.89\times 10^{-16}\;kg/s\left(\alpha =0.01\right),$ $\kappa \approx 3.43\times 10^{-6}\;N/m$.

**Figure 2:**
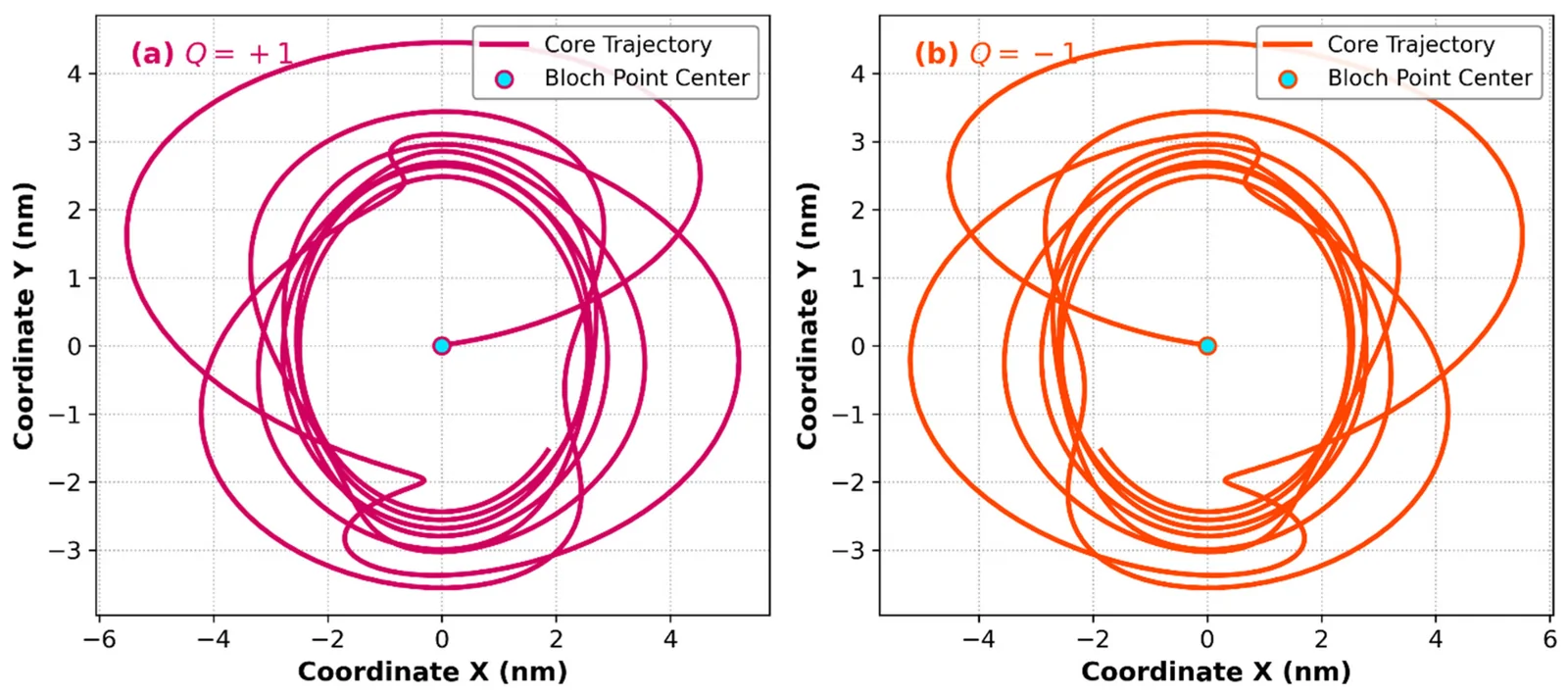
Simulated dynamic trajectories of the Bloch point (BP) core in the XY plane under a transient in-plane magnetic field pulse H_x_(t) for opposite topological charges: (**a**) Q = +1 and (**b**) Q = −1. The distinct handedness, clockwise (CW) precession for Q = +1 and counter-clockwise (CCW) precession for Q = −1, demonstrates the topological dependence of the core dynamics.

The calculated trajectories of the BP core are shown in Figure 2. For a positive topological charge (Q = +1), the gyrotropic force drives the core into a clockwise (CW) spiral precession within the parabolic pinning potential before it relaxes toward equilibrium. Conversely, for a negative charge (Q = −1), the core trajectory mirrors this behavior, precessing in a counter-clockwise (CCW) direction. This distinct topological dependence demonstrates that the handedness of the dynamic trajectory is governed by the core topology, offering a direct pathway for the non-destructive electrical identification of 3D spin textures.

### 4.2. Dynamically Generated Transverse Currents and Frequency Spectrum Analysis

The dynamic displacement of the Bloch point core induces local variations in the magnetization configuration over time, which acts as a source for spin-polarized transport and subsequently generates measurable electrical signals via dynamic spin-to-charge conversion. At the nanoscale, the time-varying spin texture pumps a spin current into the adjacent layers, where strong spin–orbit coupling facilitates its conversion into a macroscopic transverse charge current [33,43]. Such layer architecture inherently introduces an interfacial Dzyaloshinskii–Moriya interaction (DMI), which plays a role in stabilizing the underlying 3D magnetic singularity within the optimized multilayer system [44]. The general form of the current density is

(13) $$j=\theta_{SH}\frac{2e}{\hbar }\lambda_{sd}g^{↑↓}\left[M\times \frac{dM}{dt}\right]\times n$$

where *θ_SH_* is the spin Hall angle of the heavy-metal layer (or the effective spin–orbit coupling parameter), *λ_sd_* is the spin diffusion length, $g^{↑↓}$ is the interfacial spin-mixing conductance, *e* is the elementary charge, ℏ is the reduced Planck constant and ***n*** is the unit interface normal vector. Equation (13) represents the standard adiabatic spin-pumping expression derived within the scattering-matrix formalism [45].

Using the rigid-core approximation justified in Section 4.1, the entire time dependence of the magnetization is governed by the translational collective coordinate ***R***(t) = (X(t),Y(t)). Applying the chain rule, the time derivative of magnetization transforms into spatial gradients weighted by the core velocities

$$\frac{\partial m}{\partial t}=-\dot{X}\frac{\partial m_{0}}{\partial x}-\dot{Y}\frac{\partial m_{0}}{\partial y}.$$

where $\dot{X},\;\dot{Y}$ are the components of the core velocity. Substituting this expression into (13), the local charge current density takes the form

(14) $$j=-\theta_{SH}\frac{2e}{\hbar }\lambda_{sd}g^{↑↓}\left[m_{0}\times \left(\dot{X}\frac{\partial m_{0}}{\partial x}+\dot{Y}\frac{\partial m_{0}}{\partial y}\right)\right]\times n.$$

Integrating the local current density over the interfacial area S yields the macroscopic measurable transverse current along the *k*-th direction $I_{c}^{\left(k\right)}\left(t\right)=\int dS{\left[j_{c}\left(r,t\right)\right]}_{k}$. Since the Bloch-point velocity is spatially uniform within the collective-coordinate approximation, it can be factored out of the spatial integral

(15) $$I_{c}^{\left(k\right)}\left(t\right)=\sum_{i}\sigma_{ik}{\dot{X}}_{i}\left(t\right).$$

where $\sigma_{ik}$ is the geometric spin-pumping coupling tensor.

(16) $$\sigma_{ik}=-\theta_{SH}\frac{2e}{\hbar }\lambda_{sd}g^{↑↓}\int_{S}\left[{\left[m_{0}\times \frac{\partial m_{0}}{\partial x_{i}}\right]}_{k}\times n_{k}\right]dS.$$

The structure of $\sigma_{ik}$ is determined by the symmetry of the Ploch point. For an equilibrium Bloch point centered in the film, the head-to-head texture possesses three-dimensional radial symmetry. Consequently, the static configuration satisfies

$$\int_{S}\left[m_{0}\times \frac{\partial m_{0}}{\partial x}\right]dS=0,\;\int_{S}\left[m_{0}\times \frac{\partial m_{0}}{\partial y}\right]dS=0$$

because the integrands are odd under inversion about the core center. This implies that no net current is generated by a static BP. However, when the core is displaced, the broken symmetry gives rise to a finite net current. The diagonal components $\sigma_{xx},\;\sigma_{yy}$ vanish by symmetry, while the off-diagonal components survive, yielding

(17) $$I_{x}\left(t\right)=g_{xy}\dot{Y}\left(t\right),\;I_{y}\left(t\right)=-g_{xy}\dot{X}\left(t\right),$$

(18) $$\mathrm{with}\;g_{xy}=\theta_{SH}\frac{2e}{\hbar }\lambda_{sd}g^{↑↓}\int_{S}{\left[m_{0}\times \frac{\partial m_{0}}{\partial y}\right]}_{x}dS.$$

**Figure 3:**
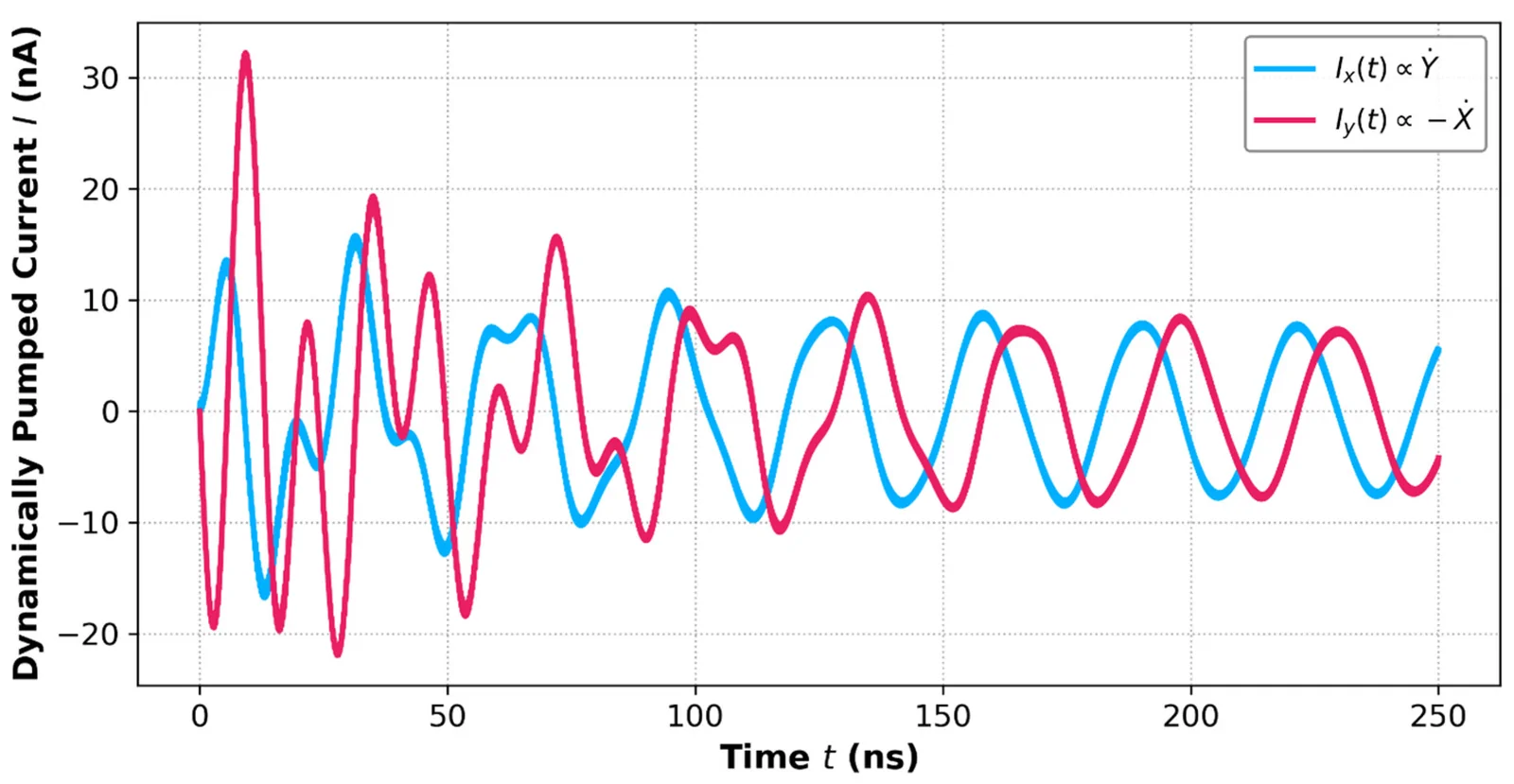
Time-dependent profiles of the dynamically generated currents *I_x_*(*t*) and *I_y_*(*t*) generated via the spin—pumping and conversion during the driven Bloch point dynamics.

This linear relation is valid within the weak-excitation regime considered here, where the spin texture undergoes rigid translational motion and higher-order corrections associated with internal deformations are negligible. Thus, the microscopic spin-pumping expression is reduced to a macroscopic transport equation in which the measured electrical signal is directly proportional to the instantaneous velocity of the Bloch-point core. This result is central to the proposed detection mechanism: the output current reflects the core dynamics, and the proportionality coefficient $g_{xy}$ is determined entirely by the equilibrium spin texture and the material parameters. The dynamically generated currents $I_{x}\left(t\right),I_{y}\left(t\right)$, calculated for the given parameters, are shown in Figure 3.

To assess the robustness of the predicted electrical signal, we derive a compact scaling estimate. From Equations (15) and (18), and using the estimates ∣σ∣ ~ Δ^2^ (the spatial integral of the magnetization gradients is localized within the core volume) and $\left|\dot{R}\right|\;\sim \;\omega \Delta$ (the core displacement scales with the core radius Δ in the linear-response regime), we obtain

(19) $$I_{c}\;\sim \;\theta_{SH}\lambda_{sd}eg^{↑↓}\Delta^{2}\omega \xi$$

where ξ ~ 0.1–1 is a dimensionless geometric factor accounting for the spatial overlap of the magnetization gradients, and ω is the characteristic driving frequency. The expression (19) reveals the following dependencies: The current exhibits linear scaling with the spin–orbit transport parameters ($\theta_{SH}\lambda_{sd}g^{↑↓}$), quadratically with the BP core radius Δ and linearly with the driving frequency ω. The damping constant *α* enters indirectly through the core velocity, with *Ic* ∝ 1/*α* in the steady-state regime. Using the baseline parameters ($\theta_{SH}\;\sim \;0.1$, $\lambda_{sd}\;\sim \;1.5\;\mathrm{nm}$ for Platinum (Pt), $M_{s}=50\;\mathrm{kA}/m$, $g^{↑↓}=10^{19}{\;m}^{-2},\;\Delta =10\;\mathrm{nm},\;\xi =0.5$), Equation (19) yields *Ic* ~ 20 nA, consistent with the numerical simulations presented below. For heavy metals with larger spin–orbit coupling ($\theta_{SH}\;\sim \;0.35÷1$), the current can reach 0.1–1 μA. Considering typical experimental data $\theta_{SH}\;\sim \;0.1\pm 0.02$, $\lambda_{sd}\;\sim \;1.5\pm 0.5\;\mathrm{nm}$, α=0.01±0.005nm, Δ=10±2 nm, the current amplitude varies within the range 5–80 nA for a Pt-based heterostructure. Even at the lower bound, the signal remains within the detection limit of modern low-noise electronics. Thus, the predicted readout mechanism is robust across a broad range of realistic experimental conditions. The quadratic sensitivity to Δ implies that multilayer designs producing larger BP radii can significantly enhance the electrical output, offering a clear pathway for signal optimization. Although direct time-resolved electrical detection of an isolated 3D Bloch point remains an experimental milestone, our estimated signal levels (10–45 nA) and GHz frequency range closely match recent experimental spin-pumping and inverse spin Hall effect measurements reported for ferrimagnet/heavy-metal interfaces under dynamic precession, supporting the experimental feasibility of the proposed readout setup.

**Figure 4:**
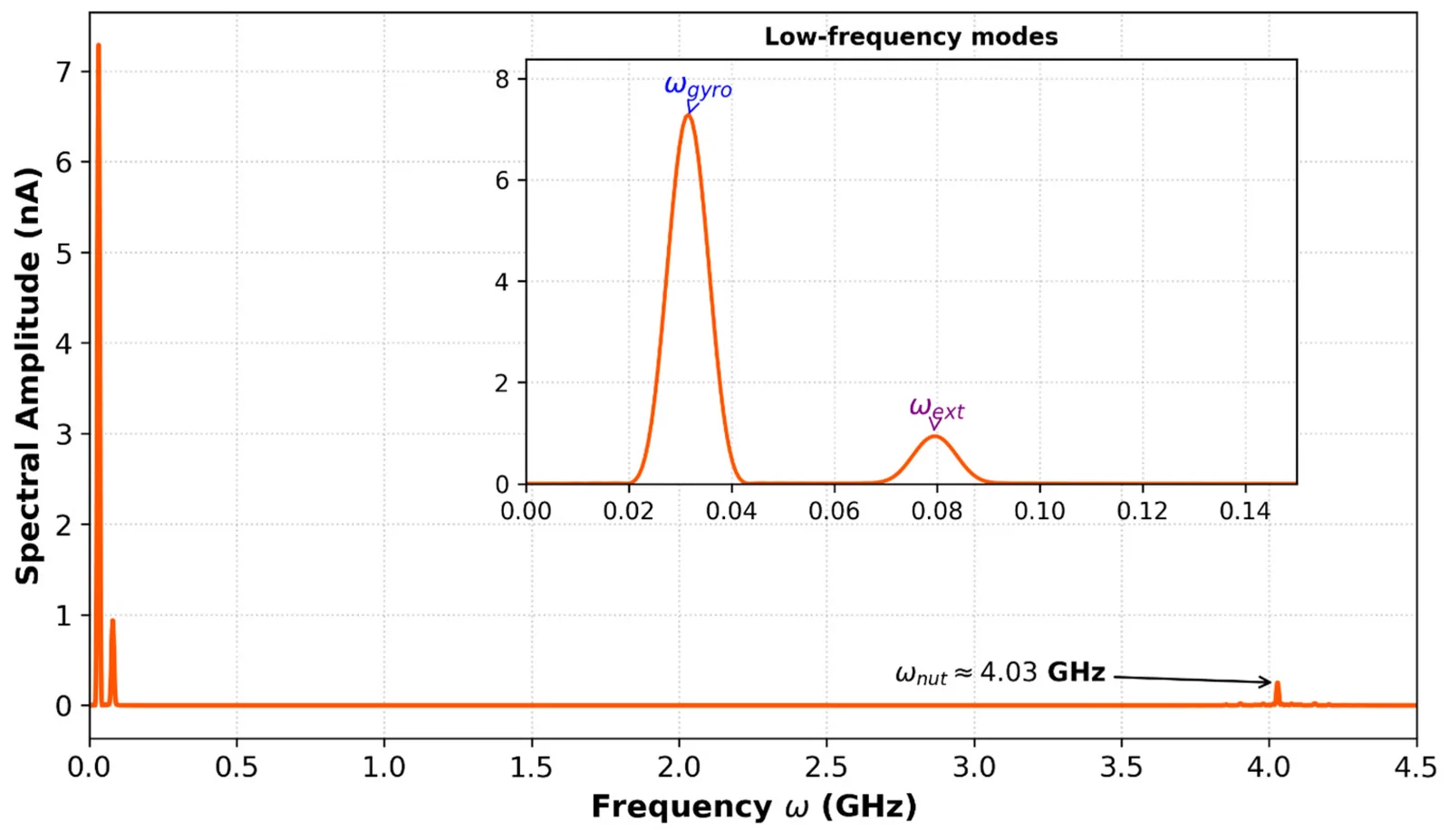
Fast Fourier Transform (FFT) amplitude spectrum of the dynamically generated current I_x_(t) revealing distinct dynamic modes of the system. The spectrum resolves three isolated harmonic contributions: the intrinsic low-frequency gyrotropic resonance mode, the external forced oscillation mode matching the driving pulse frequency, and a high-frequency peak in the gigahertz regime corresponding to the intrinsic inertial nutation of the 3D Bloch point core. Inset: Enlarged view of the 0–0.1 GHz frequency range of the FFT spectrum, showing the resolved peaks at ω_gyro_ and ω_ext_.

The complex, multi-frequency amplitude modulation observed in the current waveforms visually reflects the subtle interplay between the high-frequency inertial nutations and the lower-frequency precessional movement of the core within the magnetic trap. To decipher the exact physical mechanisms contributing to the generated electrical signal, a Fast Fourier Transform (FFT) analysis of the *I_x_*(*t*) response was performed. The resulting clear amplitude spectrum, shown in Figure 4, reveals three well-resolved, isolated harmonic contributions that match the distinct physical modes of the driven system. The first low-frequency peak ω_g_ ~ 0.02 GHz corresponds to the intrinsic gyrotropic resonance mode, which represents the natural precession of the core inside the parabolic potential well under the constraint of the gyroconstant G_0_. The adjacent low-frequency peak ω_ext_ ~ 0.08 GHz represents a forced oscillation mode that precisely mirrors the driving frequency of the external in-plane magnetic pulse. Crucially, the spectrum features a prominent high-frequency peak situated deep in the gigahertz regime ω_nut_ ~ 4.03 GHz. This high-frequency component is identified as the intrinsic nutation mode of the Bloch point, whose frequency scales inversely with the defect’s inertial property according to the relation $\omega_{nut}\;\sim \;\frac{G_{0}}{\pi m^{*}}$. The robustness of the inertial resonance was further examined within the collective-coordinate model. In the linear-response regime, the nutation frequency is determined by the intrinsic parameters of the equation of motion, primarily the effective mass m*, gyroconstant *G*, and confinement stiffness *κ*, and is therefore independent of the excitation amplitude and damping. Material and geometrical parameters (exchange stiffness, anisotropy, and layer thickness) influence the resonance indirectly through their effect on the equilibrium Bloch-point structure, shifting the frequency via corresponding changes in m* and the restoring potential. Consequently, the co-existence of intrinsic gyrotropic, forced, and nutational modulations in the output current demonstrates that the transport response reflects the fundamental mechanical properties of the Bloch point, validating these topological structures as promising candidates for frequency-tunable spintronic signal generators.

## 5. Conclusions

In summary, we have developed a theoretical and numerical framework that describes the high-frequency dynamic current response of a 3D Bloch point singularity. Utilizing a stable micromagnetic Bloch point state as the physical platform, we demonstrated that a perpendicular magnetic field effectively stabilizes the head-to-head spin defect and provides a harmonic trapping potential. Under a dynamic in-plane magnetic perturbation, the collective coordinates of the core are driven into complex, cycloidal trajectories where the particle-like effective inertial mass induces rapid nutational oscillations superimposed onto the slower gyrotropic precession.

Our analytical derivation based on the rigid-shift approximation establishes a direct connection between the microscopic spin pumping density and the macroscopically measurable transverse electrical signals, validating the cross-coupled transport relations where the output currents are directly proportional to the core velocities. The resulting output current spectrum exhibits distinct harmonic signatures, corresponding to the forced excitation, the intrinsic gyrotropic frequency, and the mass-dependent nutation peak deep in the gigahertz regime (ω_nut_ ~ 4.03 GHz). Quantitative evaluation under realistic experimental parameters confirms that peak currents of 10 to 45 nA are achievable at the interface with a heavy metal layer. Future research perspectives include exploring finite-temperature thermal fluctuations via stochastic LLG approaches, investigating non-linear and multi-frequency driving regimes, such as elliptically or circularly polarized fields, for enhanced frequency tunability, and experimentally demonstrating frequency-tunable Bloch-point nano-oscillators in multi-layered spintronic circuits. This study highlights the utility of dynamically generated currents for the non-destructive electrical detection of complex 3D topological magnetic textures, offering a viable solution to the long-standing problem of 3D spin readout and opening new pathways for high-frequency, frequency-tunable spintronic devices.

## Data Availability

The original contributions presented in this study are included in the article. Further inquiries can be directed to the corresponding author.

## Appendix A. Applicability of the Collective-Coordinate Formalism for 3D Bloch Points

The derivation presented in Section 3 relies on the collective-coordinate approximation, whose applicability to three-dimensional Bloch points deserves additional discussion. In this framework, the collective coordinates $R\left(t\right)=\left(X\left(t\right),Y\left(t\right),0\right)$ do not represent the mathematical singularity itself, but describe the displacement of the equilibrium non-collinear magnetization texture surrounding the Bloch-point core.

The validity of the rigid-translation model relies on three main assumptions: topological conservation, small displacements, and separation of frequency scales. Namely, it is assumed that (i) the topological structure of the Bloch point is preserved throughout the motion, i.e., no nucleation, annihilation, or topological transformations occur; (ii) the displacement remains small compared to the characteristic confinement length, ensuring that the pinning potential is well approximated by a harmonic function and nonlinear profile distortions remain negligible; (iii) the characteristic excitation frequencies are well separated from the eigenfrequencies of the internal deformation modes $\omega_{exc}<<\omega_{\mathrm{int}}$, where $\omega_{\mathrm{int}}$ is the lowest internal eigenfrequency of the Bloch-point texture. It should be noted that the effective inertial mass introduced in Equation (7) does not correspond to a real excitation of internal modes with finite amplitude. Instead, it originates from the virtual deformation of the magnetization texture that accompanies accelerated translational motion. These deformation contributions are integrated out within the collective-coordinate description and appear as an effective inertial parameter.

Under these conditions, internal deformation modes are weakly excited, and the dynamic response is dominated by translational degrees of freedom. More generally, the magnetization can be expanded in terms of normal modes around the equilibrium state

(A1) $$m\left(r,t\right)=m_{0}\left(r-R\right)+\sum_{n}a_{n}(t)\eta_{n}\left(r-R\right)$$

where ***η****_n_* denote internal deformation modes with time-dependent amplitudes *a_n_*(*t*). The rigid-core approximation corresponds to neglecting independent dynamics of these internal modes, i.e., assuming *a_n_*(*t*) ~ 0 for the driven response. However, the virtual contribution of these modes associated with acceleration is retained through the effective inertial mass introduced in Equation (7). Consequently, higher-order processes, such as breathing oscillations of the Bloch-point core, magnetostriction-induced elliptic distortions, coupling to exchange spin-wave modes, and variations of local magnetization magnitude, are neglected.

The separation of time scales can be estimated quantitatively. The lowest internal excitation frequencies are determined by exchange-dominated spin-wave modes

(A2) $$\omega_{\mathrm{int}}≃\gamma \frac{2A}{M_{s}}k^{2},$$

where $k≃\frac{\pi }{L_{conf}}$ is the characteristic wavevector defined by the confinement dimension. For the material parameters of the considered multilayers, the exchange length is $l_{ex}=\sqrt{\frac{2A}{\mu_{0}M_{s}^{2}}}$ ~ 8.4 nm, a typical confinement size is $L_{conf}\;\sim \;30-50\;nm$, and the lowest internal-mode frequency lies in the range

(A3) $$\nu_{\mathrm{int}}=\frac{\omega_{\mathrm{int}}}{2\pi }≃20-50\;GHz,$$

providing a clear separation of time scales between the translational and internal dynamics. Since this estimate gives internal frequencies above the characteristic translational frequencies considered in the present work, internal deformation modes are only weakly excited during the driven dynamics. This justifies the applicability of the single-coordinate generalized Thiele formalism in the linear-response regime. Our assumptions are further supported by the recently developed regularized micromagnetic theory, where the Bloch-point singularity is removed by allowing the magnetization amplitude to vary continuously. Within this framework, a generalized Thiele equation for Bloch-point textures can be derived using the same collective-coordinate approach [46]. The present model should be regarded as an effective description of the low-energy translational dynamics of a Bloch point, where all microscopic information required for the collective parameters is provided by the equilibrium micromagnetic configuration. A direct comparison with fully dynamic micromagnetic trajectories requires a separate numerical treatment of the moving Bloch-point singularity.
